# Outpatient management of severe early OHSS by administration of GnRH antagonist in the luteal phase: an observational cohort study

**DOI:** 10.1186/1477-7827-10-69

**Published:** 2012-08-31

**Authors:** George T Lainas, Efstratios M Kolibianakis, Ioannis A Sfontouris, Ioannis Z Zorzovilis, George K Petsas, Theoni B Tarlatzi, Basil C Tarlatzis, Trifon G Lainas

**Affiliations:** 1 Eugonia Assisted Reproduction Unit, 7 Ventiri Street, 11528, Athens, Greece; 2 Unit for Human Reproduction, 1st Department of Obstetrics & Gynecology, Papageorgiou General Hospital, Medical School, Aristotle University of Thessaloniki, Ring Road, Nea Efkarpia, 56429, Thessaloniki, Greece

**Keywords:** GnRH antagonist, OHSS, Luteolysis, High risk for OHSS, PCOS

## Abstract

**Background:**

Management of established severe OHSS requires prolonged hospitalization, occasionally in intensive care units, accompanied by multiple ascites punctures, correction of intravascular fluid volume and electrolyte imbalance. The aim of the present study was to evaluate whether it is feasible to manage women with severe OHSS as outpatients by treating them with GnRH antagonists in the luteal phase.

**Methods:**

This is a single-centre, prospective, observational, cohort study. Forty patients diagnosed with severe OHSS, five days post oocyte retrieval, were managed as outpatients after administration of GnRH antagonist (0.25 mg) daily from days 5 to 8 post oocyte retrieval, combined with cryopreservation of all embryos. The primary outcome measure was the proportion of patients with severe OHSS, in whom outpatient management was not feasible.

**Results:**

11.3% (95% CI 8.3%-15.0%) of patients (40/353) developed severe early OHSS. None of the 40 patients required hospitalization following luteal antagonist administration and embryo cryopreservation. Ovarian volume, ascites, hematocrit, WBC, serum oestradiol and progesterone decreased significantly (P < 0.001) by the end of the monitoring period, indicating rapid resolution of severe OHSS.

**Conclusions:**

The current study suggests, for the first time, that successful outpatient management of severe OHSS with antagonist treatment in the luteal phase is feasible and is associated with rapid regression of the syndrome, challenging the dogma of inpatient management. The proposed management is a flexible approach that minimizes unnecessary embryo transfer cancellations in the majority (88.7%) of high risk for OHSS patients.

## Background

Ovarian hyperstimulation syndrome (OHSS) is a serious complication of ovarian stimulation in patients undergoing in-vitro fertilization (IVF) treatment, which is triggered by human chorionic gonadotrophin (hCG). There are two main clinical forms of OHSS, early and late OHSS, depending on the time of onset. Early OHSS is induced by exogenous hCG administered for final oocyte maturation, usually occurring within 3–7 days post hCG [1,2]. Late OHSS is pregnancy-induced, occurs 12–17 days post hCG and is triggered by the endogenous hCG produced by an implanting blastocyst [1,2]. OHSS is further distinguished in mild, moderate and severe forms, depending on the severity of symptoms [3]. Mild OHSS lacks clinical significance, moderate OHSS requires careful patient monitoring, while severe OHSS may prove to be critical or even life-threatening, characterized by massive ovarian enlargement, ascites, pleural effusion, oliguria, haemoconcentration, adult respiratory distress syndrome and thromboembolic phenomena, and may require hospitalization in an intensive care unit [4,5].

Severe OHSS, although infrequent in the general IVF population, represents a really difficult situation for both patients and physicians. In high risk patients [6], the published incidence of severe OHSS after ovarian stimulation for IVF ranges from 10% to 38% [7-9]. This high variation in the occurrence of OHSS is mainly due to the lack of a universally accepted criteria for diagnosis and classification of OHSS [3,10].

It has been reported that in high-risk for OHSS PCOS patients, the use of GnRH antagonists is associated with a significantly decreased incidence of OHSS by 20% compared to the use of the long GnRH agonist protocol [11]. This observation was confirmed by a recent Cochrane Review [12], which reported a significant reduction in severe OHSS using the GnRH antagonist protocol in both PCOS patients and the general IVF population. However, the use of GnRH antagonists may only decrease the incidence of OHSS, which can still occur. According to the concept of an OHSS-free clinic [13], it has been proposed to trigger final oocyte maturation by replacing hCG with GnRH agonist in antagonist protocols, which appears to totally prevent the syndrome [14]. However, following GnRH agonist triggering, embryo transfer in the same cycle is associated with a significantly lower probability of pregnancy and therefore, embryo cryopreservation and transfer in a subsequent frozen-thawed cycle is usually performed [14,15]. Despite the availability of agonist triggering in antagonist protocols, some patients at high risk for OHSS will still choose to proceed to oocyte retrieval and embryo transfer using a lower dose of hCG to trigger final oocyte maturation [16] and a proportion of them will eventually develop OHSS.

It should be emphasized that in patients downregulated with GnRH agonists, which currently represent the majority of IVF patients, hCG is the only way available for triggering final oocyte maturation and thus OHSS is more likely to occur.

Alternatively, the dopamine agonist cabergoline [17], and more recently quinagolide [18], have been shown to reduce the incidence and severity of OHSS [17-21].

Currently, despite an extensive list of available prevention methods [6], if severe OHSS occurs there is no established way of management apart from conservative treatment, involving correction of fluid and electrolyte imbalance, prevention of thromboembolism, aspiration of the ascitic fluid etc. [5].

Recently, it has been suggested that GnRH antagonist administration in the luteal phase in patients with established severe early OHSS appears to prevent patient hospitalization and to result in quick regression of the syndrome on an outpatient basis. This intervention appears to be effective in both agonist and antagonist-treated patients. However, the existing published data are three small case series [22-24], which although promising, require further evaluation.

The aim of the present study was to evaluate in a larger series of patients whether it is feasible to manage women with established severe early OHSS as outpatients by treating them with GnRH antagonists in the luteal phase and cryopreserving all their embryos.

## Methods

### Study setting and patient population

This is a prospective observational, cohort study of IVF patients at high risk for OHSS, performed between January 2007 and December 2010 at Eugonia private Assisted Reproduction Unit. High risk for OHSS was defined as the presence of at least 20 follicles ≥11 mm on the day of triggering of final oocyte maturation [25].

During the study period (2007–2010) 2268 cycles were performed. Clinical and ongoing pregnancy rates were 57.2% and 49.4% respectively for patients <35 years old, 48.6% and 37.9% respectively for patients 35–39 years old, and 21.9% and 13.3% respectively for patients >40 years old. Overall severe early OHSS incidence was 1.76% (95% CI 1.28%-2.37%) (40/2268) and late OHSS incidence was 0.18% (95% CI 0.06%-0.43%) (4/2268).

### Patient management

Patients were fully explained on the last day of stimulation the possible risks in case severe OHSS developed and were presented with the following options: (a) to withhold hCG and cancel the cycle, b) to use GnRH agonist instead of hCG for triggering final oocyte maturation [14] combined with cryopreservation of all embryos , in case GnRH antagonists had been used to suppress premature LH surge, and (c) to proceed at least to oocyte retrieval using 5000 IU hCG for triggering final oocyte maturation [16] and potentially to embryo transfer, if OHSS did not occur. In the latter case, if severe OHSS occurred, embryo cryopreservation was performed and patients were treated with GnRH antagonists in the luteal phase, if possible in an outpatient setting.

All patients were monitored on days 3 and day 5 post oocyte retrieval for development of severe OHSS. In patients who did not develop severe OHSS, embryo transfer was performed either on day 3 or day 5 post oocyte retrieval according to the number and the quality of the embryos available.

The study was approved by the institutional ethics review board of Eugonia Assisted Reproduction Unit. A signed informed consent was obtained from all patients included in this study.

### Criteria for diagnosis of severe OHSS

Diagnosis of severe OHSS at our Unit is a modification based on previously published classification systems [4,6,11,17,26-29]. Severe early OHSS was diagnosed in the presence of moderate /marked ascites [10] and at least two of the following criteria: large ovaries (>100 mm maximum diameter) [11,23], haematocrit (Ht) >45%, white blood cell count (WBC) >15,000/mm^3^, hydrothorax, dyspnea, oliguria or abnormal liver function tests. Our classification criteria for OHSS include numerical parameters, useful for accurate comparisons. The existence of ascites was observed when the patient was in anti (reverse) trendelenburg position.

The classification of ascites used in our Unit (Table 1) is similar to previously published criteria [6,29] and distinguishes different levels of ascites, depending on the accumulation of ascetic fluid. This classification system is reproducible and more clear compared to the subjective description “clinical or ultrasound evidence of ascites” mentioned in previous OHSS classifications [4,26-28,30].

**Table 1:** Classification of ascites used in our Unit

**Grade**	**Description**
**No ascites**	No presence of fluid
**Low**	Small amount of fluid, barely detectable by ultrasound in the pouch of Douglas
**Moderate**	Increased amount of fluid located in the small pelvis
**Marked**	Large amount of fluid reaching the level of the umbilicus
**Massive**	Significant accumulation of fluid reaching Morrison’s pouch
**Tense**	Significant accumulation of fluid up to the level of the diaphragm with/without hydrothorax

The classification of ascites used in our Unit is similar to previously published criteria [6,29] and distinguishes different levels of ascites, depending on the accumulation of ascetic fluid when the patient was at the anti-trendelenburg position. This classification system is reproducible and more clear compared to the subjective description “clinical or ultrasound evidence of ascites” mentioned in previous OHSS classifications [4,26-28,30].

### Description of the intervention performed

In patients with established early severe OHSS on day 5 post oocyte retrieval, blastocyst cryopreservation was performed on the same day, and 0.25 mg of the GnRH antagonist ganirelix (Orgalutran, Organon, The Netherlands) was administered daily, from day 5 until and including day 8 post oocyte retrieval, as previously described [22-24].

None of the 362 patients, including the 40 patients diagnosed with severe early OHSS, received cortisone administration [31], fluid paracentesis or coasting.

In addition, in these patients daily 4500 anti-Xa IU (0.45 ml) tinzaparin sodium (Innohep; LEO Pharmacutica Products Hellas Ltd, Greece) were administered for thromboprophylaxis, from day 5 post oocyte retrieval until resolution of the syndrome.

### Follow up of OHSS

The follow-up of patients with severe OHSS following GnRH antagonist administration in the luteal phase included evaluation of hematocrit, white blood cell count (WBC) and ultrasound assessment of ovarian size and ascitic fluid on days 7, 9 and 11 post oocyte retrieval. Oestradiol and progesterone concentrations were monitored during the same period. Ovarian volume was calculated using the prolate ellipsoid formula V = D1xD2xD3x0.523, where D1, D2 and D3 are the three maximal longitudinal, antero-posterior and transverse diameters respectively.

Patients who did not develop severe OHSS and proceeded to embryo transfer were examined again 15 and 30 days after oocyte retrieval for the presence of late pregnancy-induced OHSS.

### Ovarian stimulation

Patients underwent ovarian stimulation for IVF/ICSI using either a long GnRH agonist downregulation or a flexible GnRH antagonist protocol (Lainas et al. 2010). All patients received oral contraceptive pill (OCP) starting on day 2 of spontaneous menses of the cycle prior to the treatment cycle, after blood test confirmed the presence of a baseline hormone profile. The OCP contained 0.03 mg ethinyl estradiol (E_2_) and 0.05 mg gestodene (Trigynera, Bayer Hellas, Greece). OCPs were taken daily for 21 days.

In the long GnRH agonist downregulation protocol, patients were administered s.c. GnRH agonist 0.1 mg triptorelin (Arvekap, Ipsen, France) daily. The agonist was started 3 days before discontinuation of the oral contraceptive. All patients had blood loss after discontinuation of the OCP. When desensitization was achieved (approximately 10–15 days after the initiation of GnRH agonists), as evidenced by plasma E_2_ levels of ≤50 pg/ml, the absence of ovarian follicles and endometrial thickness ≤ 6 mm) on transvaginal ultrasound examination, daily s.c. injection of recombinant FSH (rFSH; Puregon, Organon, The Netherlands or Gonal-F, Merck-Serono, Switzerland) was commenced. The dose of GnRH agonist was decreased on that day to 0.05 mg/day and continued until and including the day of triggering of final oocyte maturation.

In the flexible GnRH antagonist protocol patients started daily recombinant FSH (rFSH) treatment with s.c injections of follitropin b (Puregon, Organon, The Netherlands, or Gonal-F, Merck-Serono, Switzerland), on day 2 or day 3 of cycle that followed the discontinuation of the OCP. All patients had blood loss after discontinuation of the OCP. Daily s.c administration of 0.25 mg ganirelix (Orgalutran, Organon, The Netherlands) or 0.25 mg cetrorelix (Merck-Serono, Switzerland) was initiated when at least one of the following criteria were fulfilled: (i) the presence of at least one follicle measuring >14 mm; (ii) serum E2 levels >600 pg/ml; and (iii) serum LH levels >10 IU/l. Treatment with rFSH and GnRH antagonist continued daily thereafter, until and including the day of triggering of final oocyte maturation.

The starting dose of rFSH was 150 IU/day for all patients. This dose was adjusted after Day 5 of stimulation, depending on the ovarian response, as assessed by E2 levels and ultrasound.

### Triggering of final oocyte maturation and in vitro fertilization

Final oocyte maturation was triggered when at least three follicles of diameter ≥17 mm were present. In patients who chose to proceed to oocyte retrieval and potentially embryo transfer, 5000 IU hCG (Pregnyl; Organon, The Netherlands) were administrated i.m. In patients who chose to trigger final oocyte maturation with GnRH agonists 0.2 mg of triptorelin (Arvekap, Ipsen, France) was injected i.m. Transvaginal ultrasound-guided oocyte retrieval was performed 36 h later by double lumen needle aspiration. ICSI was performed only in cases with severe male factor or previous fertilization failure. Embryos were cultured in sequential media (Medicult/Origio, Denmark) for up to five days.

### Ultrasound and laboratory assays

All ultrasound measurements were performed using a 7.5 or 6 or 5 MHz vaginal probe (Sonoline Adara, Siemens). FSH, LH, E_2_ and P levels were measured using an Immulite analyser and commercially available kits (DPC, Los Angeles, CA). Analytical sensitivity were 0.1 mIU/ml for FSH, 0.1 mIU/ml for LH, 15 pg/ml for E_2_ and 0.2 ng/ml for P. Intra- and inter-assay precision at the concentrations of most relevance to the current study (expressed as coefficients of variation) were 2.6 and 5.8% for FSH, 5.9 and 8.1% for LH, 6.3 and 6.4% for E2 and 7.9 and 10% for progesterone. Hematocrit and white blood cell count were determined by flow cytometry using Coulter A^C^.T diff^TM^ Analyzer (Coulter Corporation, Miami, Florida). Coefficient of variation, specifying imprecision limits for white (WBC) and red blood cell count (RBC), was 3%. Hematocrit was computed from the relative volume of erythrocytes (MCV) [Ht(%) = RBCxMCV/10].

### Outcome measures

The primary outcome measure was the proportion of patients with severe early OHSS, in whom outpatient management was not feasible. Reasons for failure of outpatient management included: development of thrombosis, dyspnoea or tachypnea, severe abdominal pain or peritoneal signs, intractable nausea and vomiting that prevented ingestion of food or adequate fluids, severe oliguria or anuria, tense ascites, hypotension (relative to baseline), abnormal liver function tests, electrolyte imbalances, dizziness, or syncope [32]. Patients were instructed to contact the doctors immediately in case they fell unwell during the monitoring period for immediate admission to hospital.

Secondary outcome measures included evaluation of changes in ovarian volume, ascites volume, hematocrit values and white blood cell count, which reflect progress or regression of severe OHSS. Moreover, serum oestradiol and progesterone levels were assessed following GnRH antagonist administration in the luteal phase.

### Statistical analysis

The secondary outcome measures were subjected to repeated measures ANOVA followed by post-hoc pairwise comparisons with Bonferroni correction. The frequency distributions of the ascites levels were subjected to the chi-square test. The level of significance was set at 0.05.

Since it was impossible to know in advance the number of high risk women from the initial cohort who would develop OHSS and receive the intervention, a post-hoc power analysis was conducted. In this case, analysis involved determining the power of the patient number who received the intervention (n = 40) in the significant reduction of all the measured parameters (ovarian volume, hematocrit, WBC, estradiol, progesterone) from day 5 to day 7. It was found that in all cases the observed power of the analysis was higher that 90%, provided that a power of 80% is considered satisfactory.

## Results

**Figure 1:**
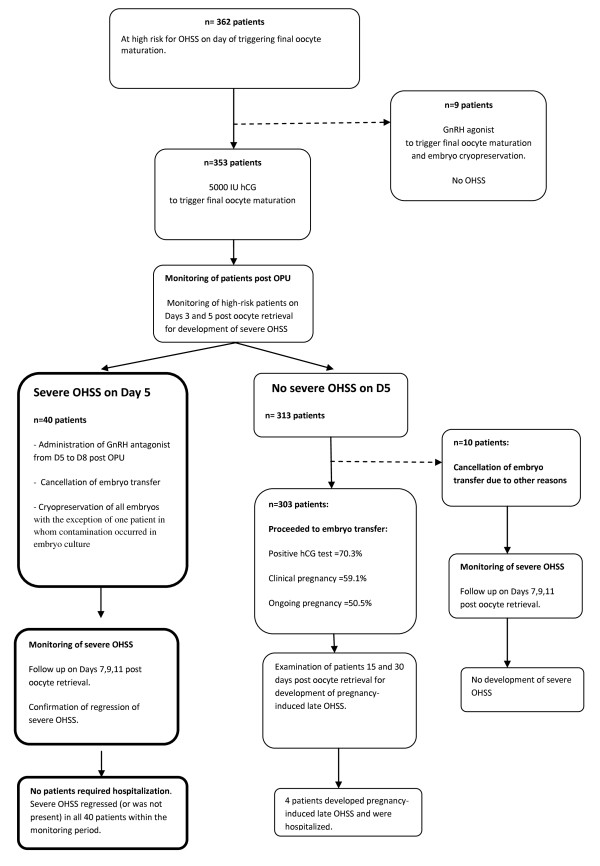
**Flow chart describing the management approach for patients at high risk for developing OHSS.** 10 patients did not develop severe OHSS and had embryo transfer cancellation due to the following reasons: failed fertilization n = 1, oocyte cryopreservation n = 2, no blastocyst formation n = 2, embryo cryopreservation due to Rokitansky syndrome n = 1, due to poor endometrium n = 1, absence of sperm on day of oocyte retrieval and use of donor sperm n = 3. Four patients with positive hCG following ET developed pregnancy-induced late OHSS and were admitted to the hospital (1.1%, 95% CI: 0.5 to 3.3).

From the cohort of 362 high risk patients included in the present study, triggering of final oocyte maturation was performed by administration of 5000 IU of hCG in 353 patients, or by administration of GnRH agonist in 9 patients (Figure 1).

Baseline characteristics of the 353 patients in whom hCG was injected for triggering final oocyte maturation and either developed (n = 40) or did not develop (n = 313) severe early OHSS are shown in Table 2, while ovarian stimulation and embryological data are shown in Table 3.

**Table 2:** Baseline characteristics for the high risk patients who injected hCG (n = 353) and either developed (n = 40) or did not develop severe OHSS (n = 313)

	**N = 313 patients without severe OHSS**	**N = 40 patients with severe OHSS**	**p**
Age (years)	32.6 ± 4.4	32.7 ± 4.0	0.960
BMI (kg/m^2^)	23.8 ± 4.8	24.8 ± 5.2	0.896
Duration of infertility (years)	3.8 ± 3.8	10.6 ± 1.4	0.958
Number of previous IVF attempts	1.1 ± 1.8	1.18 ± 1.93	0.084
Baseline FSH (IU/l)	6.7 ± 1.6	5.3 ± 1.4	**0.008**
Baseline LH(IU/l)	5.4 ± 2.5	5.6 ± 2.2	0.229
Baseline oestradiol (pg/ml)	32.9 ± 14.5	51.8 ± 27.0	0.652
Baseline progesterone (ng/ml)	0.51 ± 0.28	0.45 ± 0.23	0.157
Baseline TSH (mIU/ml)	1.7 ± 0.99	1.71 ± 0.92	0.763
Baseline prolactin (ng/ml)	16.7 ± 11.3	16.2 ± 10.5	0.566

Values are expressed as mean ± standard deviation (SD) unless otherwise stated. P-values in bold depict statistical significance (p < 0.05).

**Table 3:** Ovarian stimulation and embryological data for the high risk patients who injected hCG (n = 353) and either developed (n = 40) or did not develop severe OHSS (n = 313)

	**N = 313 patients without severe OHSS**	**N = 40 patients with severe OHSS**	**p**
Long protocol (n)	111	16	
Antagonist protocol (n)	202	24	
Duration of stimulation (days)	10.9 **±** 1.5	10.6 **±** 1.4	0.220
Total FSH (IU)	1909 **±** 636	1890 **±** 740	**0.014**
Number of follicles on day of hCG	29.0 **±** 4.4	33.8 **±** 7.0	**<0.001**
Oestradiol on day of hCG (pg/ml)	2956 **±** 1289	3687 **±** 1450	**<0.001**
Progesterone on day of hCG (ng/ml)	0.99 **±** 0.44	1.1 **±** 0.46	0.183
Number of oocytes retrieved	24.1 **±** 6.1	32.0 **±** 11.0	**<0.001**
Mature oocytes (in ICSI patients)	14.8 **±** 6.6	20.7 **±** 12.7	**<0.001**
Type of fertilization (IVF/ICSI/ IVF + ICSI)	72/160 /81	13/15/12	0.236
Number of 2PN	13.5 **±** 10.3	18.4 **±** 9.1	**<0.001**
Number of embryo transfers performed	303		
Day of embryo transfer (Day3/Day5)	131/172		
Number of embryos transferred	2.7 **±** 0.6		

Values are expressed as mean ± standard deviation (SD) unless otherwise stated. P-values in bold depict statistical significance (p < 0.05).

Patients who developed severe early OHSS had similar baseline characteristics compared to patients who did not develop severe early OHSS, apart from a significantly lower baseline FSH (p = 0.008) (Table 2).

Regarding ovarian stimulation and embryological data, patients who developed severe early OHSS had lower total FSH dose (p = 0.014), higher number of follicles (p < 0.001) and higher oestradiol levels (p < 0.001) on the day of triggering final oocyte maturation, higher number of oocytes retrieved (p < 0.001), higher number of mature oocytes (p < 0.001), and higher number of fertilized oocytes (p < 0.001) compared to patients who did not develop severe early OHSS (Table 3).

The proportion of patients at high risk for OHSS who developed severe early OHSS was 11.3% (95% CI 8.3%-15.0%) (40/353). Incidence of late OHSS in patients at high risk who were triggered with hCG, did not develop early OHSS and proceeded to embryo transfer was 1.1% (95% CI 0.36%-2.71%) (4/353) and was associated with pregnancy achievement.

All 40 patients with severe early OHSS had blastocyst cryopreservation in combination with GnRH antagonist administration.

**Figure 2:**
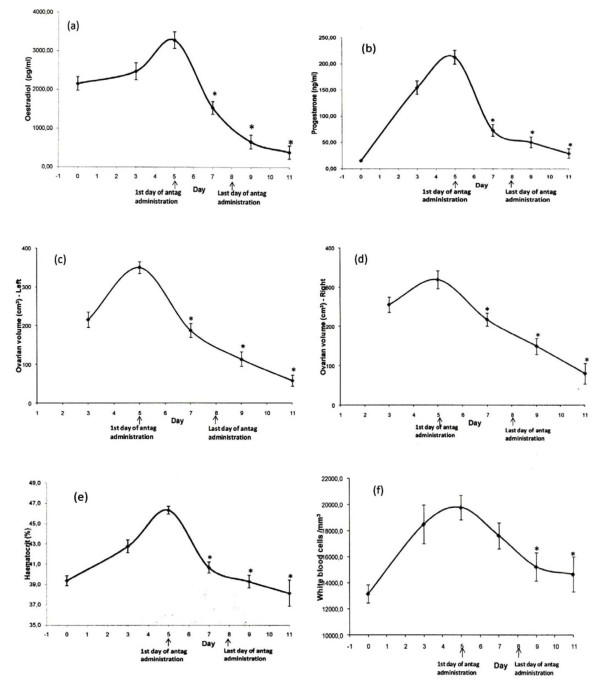
**Concentrations of (a) oestradiol (b) progesterone, (c) left and (d) right ovarian volume, (e) hematocrit, and (f) white blood cells during the monitoring period.** Asterisks depict statistically significant difference compared to day 5 (*P < 0.001). Oocyte retrieval was performed on day 0. GnRH antagonist was administered from day 5 until and including day 8 post oocyte retrieval, as indicated by arrows.

**Figure 3:**
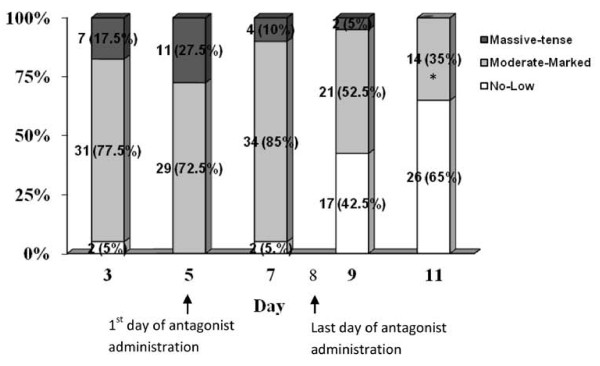
**Distribution of ascites levels during the monitoring period.** *At the end of the monitoring period, on day 11, “moderate-marked ascites” included 14 women (35%), of which 11 (27.5%) had moderate ascites, and only 3 (7.5%) had marked ascites. No women (0%) had “massive-tense” ascites, while the majority of women (65%) had no or low levels of ascites on day 11 of monitoring (chi-square p < 0.01).

In all 40 patients with severe early OHSS outpatient management was feasible and none required hospitalization following administration of GnRH antagonist 5 days after oocyte retrieval and embryo cryopreservation (0%, 95% CI: 0 to 8.8). Patient monitoring showed improvement of patients’ symptoms, ultrasound and laboratory findings (Figures 2, 3).

In the 40 patients with severe early OHSS diagnosed on day 5 post oocyte retrieval, the highest values of hematocrit, WBC count, ovarian volume, as well as oestradiol and progesterone were observed on the day of severe OHSS diagnosis (day 5 post oocyte retrieval) (Figure 2).

Compared to Day 5 post oocyte retrieval, hematocrit, ovarian volume, oestradiol and progesterone declined significantly (p < 0.01) two days (Day 7) after initiation of GnRH antagonist administration, while WBC count displayed a significant decrease on day 9, compared to day 5 (day of GnRH antagonist initiation) (Figure 2).

All 40 patients with severe OHSS were diagnosed with significant ascites on the day of GnRH antagonist initiation (27.5% massive/tense ascites; 72.5% moderate/marked ascites), which progressively declined to moderate levels (35%) or low/no detectable levels (65%) at the end of the monitoring period (p < 0.01) (Figure 3). Three patients (3/303) displayed marked ascites on day 11 of monitoring.

One patient with severe early OHSS developed mild respiratory problems on Day 5 post oocyte retrieval. The patient was examined by a pathologist, O_2_ saturation was checked, and it was decided that admission to hospital was not necessary at that moment. The patient was instructed to contact the doctors in case she developed dyspnoea, dizziness, fainting, or general discomfort, for immediate admission to hospital. However, the mild respiratory problem disappeared within 24 hours, along with a general improvement of clinical symptoms.

In high risk patients who did not develop severe early OHSS and proceeded to embryo transfer (n = 303), biochemical, clinical and ongoing pregnancy rates were 70.3% (n = 213), 59.1% (n = 179) and 50.5% (n = 153), respectively (Table 4).

**Table 4:** Pregnancy rates in high risk patients who did not develop severe early OHSS and proceeded to embryo transfer (n = 303)

**Pregnancy type**	**Rate**
Biochemical pregnancy n (%)	213 (70.3%)
Clinical pregnancy n (%)	179 (59.1%)
Ongoing pregnancy n (%)	153 (50.5%)

## Discussion

This is the first observational cohort study in the literature describing outpatient management of established severe early OHSS, using administration of GnRH antagonist in the luteal phase and cryopreservation of all embryos. None of the patients evaluated required hospitalization, suggesting that outpatient management is feasible using this approach. Luteal antagonist administration was associated with rapid regression of established severe early OHSS, improvement of patient symptoms and ultrasound and laboratory findings. This flexible approach allows high risk patients to safely proceed at least to oocyte retrieval using low dose hCG for triggering final oocyte maturation, and potentially to embryo transfer if severe OHSS does not occur, thus avoiding an unnecessary embryo transfer cancellation in the majority (88.7%) of high risk patients.

Administration of GnRH antagonist in the luteal phase appears to result in rapid resolution of severe OHSS as early as two days after initiation of GnRH antagonist, with a significant decline of ovarian volume, hematocrit and ascites, as well as oestradiol and progesterone concentrations, confirming previous reports published in three small case series [22-24]. This rapid decline continued in a progressive manner until the end of the monitoring period.

The rapid decrease of ovarian volume, oestradiol and progesterone levels, observed in the patients with established severe OHSS, suggests a luteolytic effect of the GnRH antagonist, as recently proposed [23,24]. It is believed that hCG administration for triggering of final oocyte maturation induces massive luteinization, elevated secretion of angiogenic factors (such as vascular endothelial growth factor, angiotensin II, interleukins, histamine, prolactin, prostaglandins, endothelin-1, selectins) from multiple corpora lutea of hyperstimulated ovaries, leading to an increase of vascular permeability, fluid shift to the third space and finally development of OHSS [33-36].The luteolysis induced by GnRH antagonist possibly leads to a decrease of ovarian activity and to minimized secretion of locally produced angiogenic factors, resulting in regression of severe OHSS.

The results obtained in the current study might be explained by a direct action of GnRH antagonist on the ovary. The presence and function of extrapituitary GnRH receptors has been demonstrated in several tissues, including the human ovary [37]. In addition GnRH antagonists have been shown to inhibit the expression of locally produced ovarian angiogenic factors, such as VEGF [38], in human granulosa luteal cell cultures.

It seems unlikely that the luteolytic action of GnRH antagonist occurs by a decrease in LH secretion, since LH concentrations following ovarian stimulation for IVF are deeply suppressed in the luteal phase [39].

It can be assumed that severe OHSS resolution observed within the monitoring period of the current study may be due to the action of GnRH antagonist rather than natural course of the syndrome. It is recognized that OHSS is a self-limited disease, which however requires an extensive period for natural regression, with prolonged hospitalization ranging from 11 to 23 days [40], occasionally in intensive care units, accompanied by multiple ascites punctures, human albumin administration, correction of intravascular fluid volume and electrolyte imbalance [32]. On the contrary, our proposed approach resulted in rapid regression of severe OHSS as early as two days after initiation of GnRH antagonist, without any use of invasive treatment for the patients and avoiding the need for hospitalization.

It has been shown that while elective embryo cryopreservation can prevent pregnancy-induced late OHSS, it cannot completely eliminate early OHSS, which is induced by exogenous administration of hCG for triggering final oocyte maturation [3,41-43].

In addition, it was recently proposed that early OHSS can be equally severe as late OHSS, requiring a mean of 6.8 to 20 days of hospitalization depending on whether the women subsequently became pregnant or not [44], despite previous reports showing that late pregnancy-induced OHSS is associated with more severe symptoms [1,2].

In the present study, only 11.3% (95% CI 8.3%-15.0%) (40/353) of high risk patients receiving hCG developed severe OHSS and required the intervention (i.e. GnRH antagonist administration and cryopreservation of all embryos). The occurrence of severe OHSS presented here is in agreement with the 12.6% rate shown for PCO patients [9], and is at the lower end (10%) of the incidence range previously reported in the literature for high risk patients (10-38%) [7,8]. In addition, the extremely low percentage (1.1%) of late OHSS in the present study is significantly lower than the 10% rate of late OHSS previously reported for women with polycystic ovaries [2], probably indicating the importance of accurate identification and monitoring of high risk patients. Using the proposed approach, it is possible to minimize the occurrence of late OHSS by accurate diagnosis of early severe OHSS prior to the performance of embryo transfer.

Despite the prevention of late OHSS by elective embryo cryopreservation shown in the present study, the possible transfer of embryos combined with luteal administration of GnRH antagonist could potentially be problematic and lead to lower implantation rates due to a putative negative effect of the antagonist in the hormonal profile to sustain implantation.

The proposed approach offers a flexible alternative solution in cases when GnRH agonist for triggering final oocyte maturation (OHSS-free clinic [13]) is not feasible, i.e. in patients treated with a long protocol or patients not accepting embryo transfer cancellation. Also, administration of GnRH antagonist in the luteal phase may prove useful in patients who develop severe OHSS despite the use of preventive measures.

## Conclusions

In conclusion, the current study suggests, for the first time, that successful outpatient management of severe OHSS with antagonist treatment in the luteal phase is feasible and is associated with rapid regression of the syndrome, challenging the dogma of inpatient management. The proposed management is a flexible approach that minimizes unnecessary embryo transfer cancellations in the majority of high risk for OHSS patients.

## Abbreviations

ANOVA: Analysis of variance; E2: Oestradiol; GnRH: Gonadotrophin-releasing hormone; FSH: Follicle stimulating hormone; hCG: Human chorionic gonadotrophin; ICSI: Intracytoplasmic sperm injection; IVF: in vitro fertilization; LH: Luteinizing hormone; OCP: Oral contraceptive pill; OHSS: Ovarian hyperstimulation syndrome; PCO: Polycystic ovaries; VEGF: Vascular endothelial growth factor; WBC: White blood cells.

## Competing interests

The authors declare that they have no competing interests.

## Authors’ contributions

GTL participated in the study design, acquisition and analysis of data and writing of the manuscript. EMK participated in the analysis and interpretation of data, writing and revision of the manuscript. IAS participated in the acquisition, analysis and interpretation of data, writing and revision of the manuscript, and performed embryology work. IZZ and GKP participated in the interpretation of data and performed clinical work. TBT and BCT participated in the interpretation of data and revision of the manuscript. TGL had the original conception and general supervision of the study, participated in study design, acquisition, analysis and interpretation of data, writing and revision of the manuscript, and performed clinical work. All authors read and approved the final manuscript.
